# Donor-derived infections in solid organ transplant recipients

**DOI:** 10.1097/MOT.0000000000001094

**Published:** 2023-08-08

**Authors:** Maddalena Peghin, Paolo Antonio Grossi

**Affiliations:** Infectious and Tropical Diseases Unit, Department of Medicine and Surgery, University of Insubria-ASST-Sette Laghi, Varese, Italy

**Keywords:** donor derived infection, emerging pathogens, HTLV I/II, monkeypox, SARS-CoV-2, transplantation

## Abstract

**Purpose of review:**

The potential for transmission of donor-derived infections (DDIs) is impossible to eliminate, but a thoughtful and systematic approach to donor evaluation can mitigate the risk. Prevention is a key issue and clinicians must maintain a high index of suspicion and remain vigilant in staying up to date on emerging infections. COVID-19 and Monkeypox have represented a new challenge for infectious disease screening and recommendations have been evolving, as knowledge in the field has grown. Additional considerations for pretransplant deceased donor screening include testing for neglected and endemic infectious diseases such as strongyloidiasis and HTLV 1/2. Molecular diagnostic tests have improved awareness on pathogenicity of mollicutes and fungi in the setting of DDIs. The aim of this review is to provide an update on the most recent literature on DDI with a special focus on these emerging hot topics.

**Recent findings:**

Donor screening for uncommon pathogens must be guided by knowledge of changing epidemiology of infectious disease and availability of new diagnostic methods.

**Summary:**

Appropriate screening, early recognition, timely reporting, close monitoring, and appropriate management are essential to help reducing the risk of emerging DDIs.

## INTRODUCTION

Expected and unexpected donor-derived infections (DDIs) remain an intrinsic risk of solid organ transplant (SOT) and, although rare, have been associated with significant morbidity and mortality. According to the most recent report of the Disease Transmission Advisory Committee unexpected DDIs were rare, occurring in 0.18% of all SOT recipients. However, graft loss or death occurred in about one-third of recipients with DDIs, with higher rates associated with parasitic and fungal diseases [1]. 

**Box 1:**
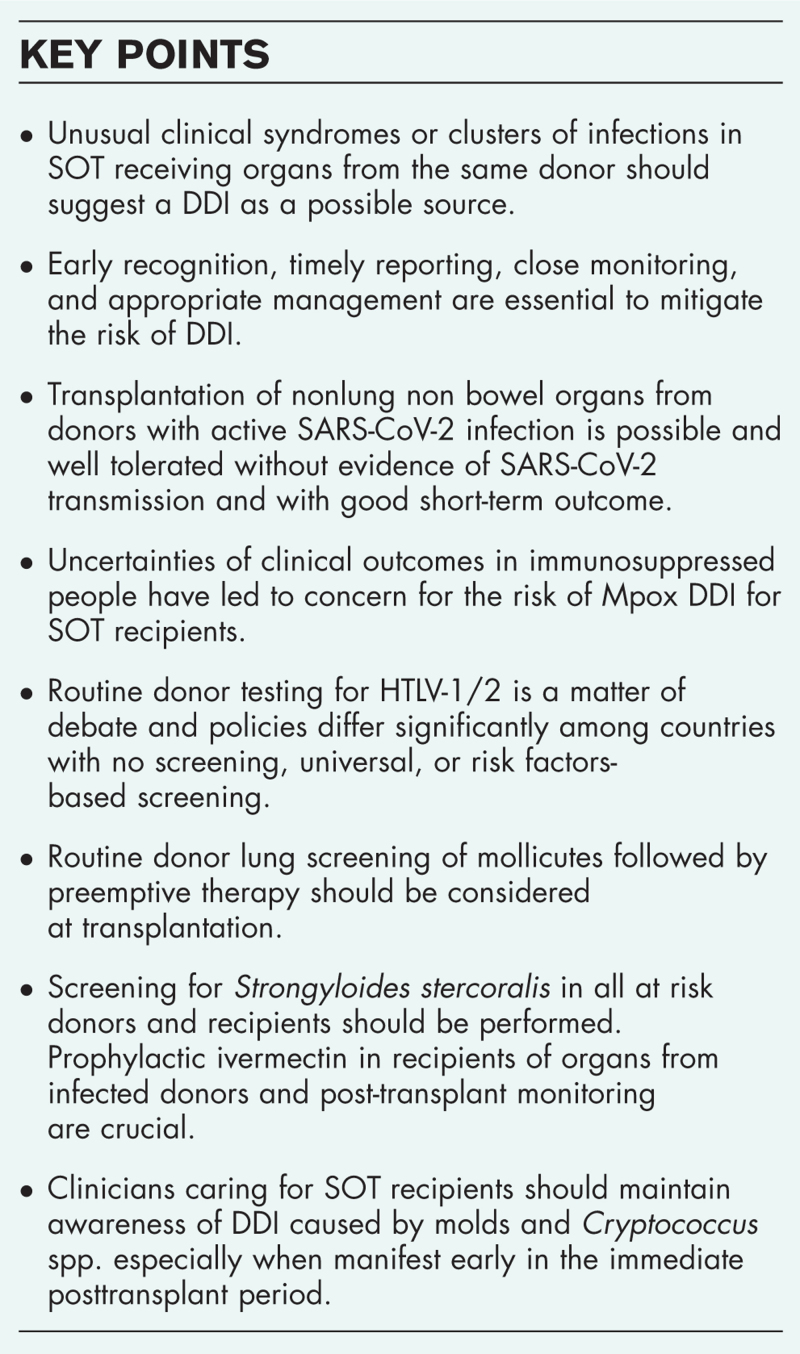
no caption available

The prevention of the transmission of infections with organ transplantation requires compliance with a well defined path that allows an adequate level of safety by simultaneously increasing the number of organs for transplantation. The mitigation risk process for DDIs should be systematic and involve interview of the next of kin regarding infectious diseases in the deceased donor, clinical and laboratory screening of at-risk donor, timely notification of DDIs to organ procurement organization and transplant centers, high index of suspicion of possible DDIs in the recipient and use of prophylactic therapy in recipients of organs from infected donor, whenever possible.

However, it is also necessary to adapt the methodological approach to specific situations regarding new epidemiological challenges which are taking advantage of the climate change and globalization and thanks to the increased microbiological testing armamentarium, which has improved diagnosis of DDIs. Therefore, donor screening for uncommon pathogens must be guided by knowledge of changing epidemiology and dynamic transmission of infectious disease. The aim of this review is to provide an update on the most recent literature on DDIs with a special focus on emerging hot topics (Table 1) [1].

**Table 1:** Summary of screening indication and management for donor-derived infection caused by emerging pathogens

	Donor pretransplant screening	Results of donor screening mandatory before transplantation	Active infection contraindicates donation	Available prophylaxis	Recommendation for universal use of prophylaxis in recipients
COVID-19	Universal	Yes	No	Yes	No
Mpox	Only if risk factors	Yes	Yes	Yes but no experience	Yes
HTLV-1/2	Only if risk factors	Yes	Yes, for seronegative recipient	No	Not available
Mollicutes	Universal for lung donation	No	No	Yes	Yes
Strongyloidiasis	Only if risk factors	No	No	Yes	Yes
Cryptococcus spp. and molds	Universal for standard cultures Individualized only if risk factors	No Individual donor evaluation required	Individual donor evaluation required	Yes	Yes

## SARS-CoV-2

At the beginning of the Coronavirus disease 2019 (COVID-19) pandemic, the speed of the disease, the overwhelm of healthcare system and uncertainties on the risk of COVID-19 transmissibility from donors to recipients significantly affected the landscape of organ donation and transplantation worldwide [2].

Early in the outbreak period, international Transplant Societies recommended screening of donors for severe acute respiratory syndrome Coronavirus 2 (SARS-CoV-2) and recommended against the use of organs from donors with active SARS-CoV-2 infection [3]. However, restrictive policies resulted in the loss of a significant number of lifesaving organs, and based on the better understanding of the biology of SARS-CoV-2 and the growing experience, recommendations have been critically questioned and reconsidered [4–6]. Thanks to the growing knowledge, there has been a significant trend toward increased use of organs from donors with resolved or active SARS-CoV-2 infection and most Transplantation Societies have issued regularly updated guidance with variable practice in acceptance and management [4,7^▪▪^].

The possibility of SARS-CoV-2 transmission through organ donation has only been reported for lung transplantation and seems it may occur possibly with intestinal donation [8]. On the basis of the current experience, transplantation of nonlung organs from donors with active SARS-CoV-2 infection is considered possible and well tolerated, without SARS-CoV-2 transmission and with evidence of good short-term outcomes, in terms of hospital length of stay, 30-day graft loss, and mortality [9^▪^,^10^,^11^].

The use of thoracic organs from COVID-19 positive donors is being explored. Potential use of tests that can predict viral infectivity, such as sub-genomic RNA testing, has been observed to be successful for lung transplantation using an allograft from a COVID-19–recovered donor [12^▪▪^]. Recent small series have shown that lungs from donors with a positive SARS-CoV-2 PCR may be successfully utilized for lung transplantation with careful donor selection, including asymptomatic status, positive nasopharyngeal (NPS) swab, negative bronchoalveolar lavage, cycle threshold values more than 34, first positive NPS COVID-19 test older than 20 days [11,13,14].

COVID-19 virus can cause subclinical endothelial dysfunction and organ injury in potential donors.

There is a lack of robust investigations evaluating the longer-term evolution of these recipients. Outcomes of kidney recipients of donors with active or resolved COVID-19 have been observed to be comparable to recipients of COVID-negative donors (all-cause graft loss, all-cause death, acute rejection, delayed graft function, hospitalization) over more than 2 years of follow-up [10,15]. Similar patient and graft survival was reported in liver transplant recipients at 1 year regardless of donor COVID-19 infection status [16]. Of note that a recent study using the Organ Procurement and Transplantation Network database found an increase in 6-month and 1-year mortality among adult heart transplant recipients from active COVID-19 donors compared with non-COVID-19 donors. However, heart transplant recipients from donors recently recovered from COVID-19 had similar survival rates to that of heart transplant recipients from non-COVID-19 donors [7^▪▪^]. Further studies with more granular clinical data are needed to assess the long-term outcomes of recipients of organs from donors with active SARS-CoV-2 infection.

## MPOX

Since May 2022, a Mpox (Monkeypox) outbreak has been reported from countries where the disease is not endemic with the highest number of cases in the USA, South America, and Europe [17] (https://worldhealthorg.shinyapps.io/mpx_global/). On July 23, 2022, the WHO declared this outbreak a public health emergency of international concern. Since then, the frequency of reported cases has decreased substantially (https://worldhealthorg.shinyapps.io/mpx_global/).

With the exception of West and Central Africa, the ongoing outbreak of Mpox continues to primarily affect MSM through sexual contact. Mortality has been observed to be low and may be associated with the rare development of severe clinical presentation, including fulminant skin lesions and systemic complications, especially in severely immunosuppressed hosts [17,18].

Uncertainties of clinical outcomes in immunosuppressed people has raised concerns about the risk of Mpox for SOT recipients [19]. Moreover, as Mpox can be transmitted by body fluids, there is a hypothetical risk of transmission from infected donors to transplant recipients. International Transplant Societies have provided guidance regarding evaluation of donors actively infected, recovering, or exposed to Mpox virus. It is not recommended to screen routinely donors without clinical features of Mpox infection. Diagnostic screening (lesion and blood PCR) is currently suggested only in donors with specific risk factors for infection and/or with compatible clinical presentation [19]. Organs from donors with confirmed, probable, or possible active Mpox should not be used based on current limited information. Confirmed Mpox-infected patients should be considered for donation when all lesions are crusted and skin re-epithelized. Individual risk–benefit discussion should be done for donor with contact with Mpox in the previous 21 days (maximum incubation period) (https://www.myast.org/monkeypox-faqs-transplant-community).

There are two available vaccines that can reduce the risk of developing Mpox and may be used for pre and postexposure prophylaxis [20]. The modified vaccinia Ankara vaccine is a nonreplicating vaccinia virus with excellent safety profile, even in immunocompromised people, that poses no risk of transmission through donor vaccination. The ACAM2000 vaccine, a replication-competent smallpox vaccine, is contraindicated in highly immunocompromised patients and may pose unlikely risk of transmission through donor vaccination [21].

As things stand now, no DDI Mpox infection has been described, but in case of transmission treatment with antiviral in monotherapy (tecovirimat) or combination treatment (tecovirimat and cidofovir/brincidofovir) may be considered. When initiating treatment, clinicians should assess for potential drug interactions between tecovirimat and immunosuppressive agents. In addition, the use of intravenous vaccinia immune globulin and vaccine as postexposure prophylaxis may be considered [20].

## HTLV 1/2

Human T-cell leukemia virus type-1 and type-2 (HTLV-1 and HTLV-2) infection is a neglected disease, despite infecting 10 million people worldwide and severe illnesses developing in 5–10% of carriers [22]. The spectrum of diseases includes adult T cell leukemia-lymphoma, associated myelopathy/tropical spastic paraparesis (HAM, TSP), and other systemic diseases. HTLV-1/2 infection has been known to be transmitted vertically between mother and child, sexually, by blood transfusion, sharing needles, and organ transplantation.

The diagnosis of HTLV-1/2 infection is generally based upon serologic testing. The most frequently used screening tests are enzyme immunoassay (EIA), ELISA, and chemiluminescence (CLIA), which may have variable predictive value depending on the population and the employed test. Western blot is usually used for confirmatory testing and distinguish between infection with HTLV-1 and the less pathogenic HTLV-2. Of interest that it has recently been suggested that a signal/cut-off (S/CO) ratio higher than the manufacturer's recommendation of 1.0 in the Abbott Architect antibody assay is a reliable measure of HTLV-1/2 infection. Interpretation of these ratios can assist clinicians in the assessment of low reactive samples and reiterates the need for faster access to confirmatory testing [23^▪^].

Routine donor testing for HTLV-1/2 is currently a matter of debate and policies differ significantly among countries with no screening, universal (UK since 2011 and Spain since July 2019) or risk factors-based screening (https://freepub.edqm.eu/publications/PUBSD-88/detail) [24–27]. On the one hand, the low number of described HTLV-1/2 DDIs and the relatively high rate of false positives routine testing for HTLV-1/2 may be associated with significant organ wastage. On the other hand, the rate of HTLV-1/2 is low but not negligible in donors/recipients of SOT depending on the epidemiological setting. HTLV-1 transmission form donors with no risk factors has been reported and transmission of HTLV-1/2 DDIs has been associated with HTLV-1/2 associated diseases (mainly TSP) in about 66% of patients [22,27,28]. Moreover, the evolution of HTLV-1/2 HAM has been observed to have a shorter period from infection to the onset of symptoms (2 months--8 years), compared with a longer period (15–20 years) in the immunocompetent individuals [29,30].

Universal screening with serology in all donors should be performed through automated, approved tests that are efficient, fast, and with an acceptable cost. Risk factors based screening is especially indicated in donors from or who have lived in endemic areas of HTLV-1/2 infection; donors who are children of mothers born or residing in endemic area; and donors, especially women, whose partners have resided in endemic areas. Regions with high endemicity of HTLV-1/2 are southwestern Japan, Caribbean area, sub-Saharan Africa, South America, and isolated foci in the Middle East and Oceania [30]. In Europe, only Romania represents an endemic area for HTLV-1/2. However, there are worldwide evolving foci of HTLV-1 infections [26].

In the case of seropositive donor and seronegative recipient, rejection of the organ is recommended. In the case of a seropositive donor and a HTLV-1/2 seropositive recipient, organ acceptance may be considered taking into account the potential lower risks of development of associated disease in those already infected [30].

Acute graft versus host disease (aGVHD) is a rare, but severe complication of liver transplantation and maybe caused by the activation of donor immune cells in the graft against the host shortly after transplantation. Of interest that a recent study showed that HTLV-1/2 infection of donor-derived T cells might promote aGVHD [31^▪▪^] in liver transplant recipients.

The debate is still ongoing; however, we believe that with the improvement of the currently available HTLV-1/2 diagnostic assays, universal screening might be introduced to minimize the risk of transplanting organs from HTLV-1-infected donors, avoiding organ wastage due to false-positive results.

## MOLLICUTES

*Ureaplasma* species (*U. parvum* or *U*. *urealyticum*) and *Mycoplasma hominis,* herein referred to as mollicutes, are bacteria that colonize the genitourinary system and the upper respiratory tract in humans.

Difficulty identifying these organisms that lack a cell wall is a significant barrier to diagnosis and special cultures, PCR screening, and further tests are required for the diagnosis.

In the postlung transplant setting, these organisms are known to cause mediastinitis, surgical site infection pericarditis, empyema, bronchial anastomosis complications, and have been associated with fatal cases of hyperammonemia syndrome, which is far more frequent in lung recipients [32,33^▪▪^,^34^].

The exact source of the *M. hominis* and *Ureaplasma* species infections is heavily debated. Pretransplant recipient urine screening had shown to have a low yield and has not been correlated with posttransplant mollicute infection. In contrast, most *M. hominis* and *Ureaplasma* species infections in lung transplant recipients have been observed to be donor-derived [35]. Routine donor lung screening for mollicutes has been found to have a prevalence of about 10%. Young age, female sex, and a history of high-risk behavior (mainly high-risk sexual practices) are well known risk factors of the donor [36].

On the basis of this growing evidence, routine lung donor screening of mollicutes followed by preemptive therapy should be considered at transplantation and has been incorporated by several international guidelines (https://freepub.edqm.eu/publications/PUBSD-88/detail). The role of antimicrobial therapy in these cases is still unclear, but in recipients from lung donors with positive screening, we recommend a treatment, which may include typically a combination of a fluoroquinolone, a tetracycline, or a macrolide antibiotic, due to increasing reports of antibiotic resistance [34–37].

## STRONGYLOIDES STERCORALIS

Strongyloidiasis is a neglected tropical disease caused by the nematode *Strongyloides stercoralis.* Infection with *S.stercoralis* is typically a chronic asymptomatic infection in immunocompetent hosts, but SOT recipients are at an increased risk for hyperinfection syndrome and/or disseminated disease, frequently resulting in fatal outcomes (up to 40–85%). Of note that concomitant bacteremia and/or meningitis is observed in 50% of cases [38].

The usual mechanism of infection in SOT recipients is reactivation of chronic *S. stercoralis* infection following immunosuppressive therapy [39]. *S. stercoralis* DDI infection in SOT recipients is rare, but recognized as an emerging infection. Due to variable clinical presentation and underdiagnosis with therapeutic delays, DDIs due to *S. stercoralis* have been associated in most series with unfavorable outcomes [1,40^▪▪^].

Previous screening recommendations have focused on preventing reactivation by testing for chronic infection in at-risk recipients, but current guidelines recommend screening program for *S. stercoralis* to be strongly considered also in donors with epidemiologic risk factors (based on country of origin and travel history) or unexplained eosinophilia [41,42]. Areas with high endemicity include Southeast Asia, Central and South America, Africa, Bangladesh, and Pakistan (estimated 10–60% incidence).

Donor testing includes stool ova and parasite exam, which may be difficult to perform and with a low yield of a single stool specimen (15–30%) and IgG ELISA antibody testing, which are more sensitive (84–95%) than conventional direct identification methods.

The results of *S. stercoralis* serology should not preclude transplantation [38]. However, timely administration of prophylaxis may be needed to prevent the consequences of possible transmission in recipients of donors with suspected or proven *S. stercoralis* infection or with a positive screening test. There is currently no consensus on prophylaxis recommendation, but two sequential days of ivermectin (200 μg/kg daily) with a second 2 days course 2 weeks later has been observed to be effective in recipients who received organs from infected donors. After prophylactic treatment, the recipient should be monitored for appearance of related signs or symptoms, due to the limited reliability of serologic testing in the setting of immunosuppression [38].

## CRYPTOCOCCUS AND MOLDS

Although rare, fungal DDIs caused by *Cryptococcus* spp. and molds have been associated with high rates of serious complications, vascular invasion, graft loss and death [1]. The high mortality may be related to delay in diagnosis due to the lack of consideration of donor exposures, variable latency period, unspecific clinical presentation in the recipient in the posttransplant period, and lack of reliable diagnostic tools.

Donor-derived cryptococcosis is a rare but severe complication of SOT (about 22% mortality). Active systemic fungal infection is a contraindication for transplantation. However, pretransplant donor screening for latent fungal infections including cryptococcosis is not routinely recommended [43]. Testing prior to donation with serum *Criptococccus* Ag or cerebrospinal fluid *Criptococccus* Ag (if suspicion is high) is suggested in donors with underlying risks for *Criptococccus* spp. such as immunocompromising conditions, unexpected central nervous system findings (aseptic meningitis), unexplained intracerebral event (stroke at a young age), or increased intracranial pressure. Late diagnosis of donor cryptococcosis after transplant or development of cryptococcosis in the recipient early after transplantation (within 1 months, maximum 1 year) is recommended to be notified to organ procurement organization and to the transplant centers to assess other recipients’ status and to evaluate implementation of prophylaxis with fluconazole [44–46].

Aspergillosis and other molds may also be transmitted from infected donors and can cause invasive fungal infections in transplant recipients with very high mortality rates (up to 50%). Of interest that aspiration of water during drowning events can expose victims to environmental molds (Mucorales and *Scedosporium*) [47^▪▪^]. Other specific clinical circumstances associated with transmission of filamentous fungi through organ donation include being a transplant recipient, brain hemorrhage as the cause of death, prolonged ICU stay, and mechanical ventilation [48]. Donor-derived graft mucormycosis mostly derive from cases developing outside of the Western world or associated with transplant tourism. In other cases, transmission is expected to derive from contamination during procurement, organ handling, or nonsterile condition [49]. Therefore, clinicians should maintain clinical suspicion for invasive mold infections and consider antifungal prophylaxis with drugs active against molds in SOT recipients receiving organs from donors with specific risk factors [47^▪▪^].

A key issue with respect to diagnosis is the need to develop sensitive and reliable assays [50]. Of note that nucleic acid tests and metagenomic next-generation sequencing is being explored and has been recently highlighted for fungi diagnosis in the setting of donor-derived transmission of scedosporiosis [51,52].

## CONCLUSION AND FUTURE DECISIONS

Donor-derived infections continue to be a challenge. The discovery of and the spread of emerging or reemerging pathogens continue. Due to the shortage of organs for transplantation, innovative screening approaches must be constantly applied to improve safety and quality of organs allocation, along with awareness of epidemiological changes. It is of paramount importance that the transplant community continues to engage in high-quality research and innovation to develop new diagnostic tools, therapies, and a more tailored approach for SOT recipients who remain at risk of old and new emerging DDIs.

## Acknowledgements

*P.G. and M.P. performed the literature research and wrote the manuscript. P.G. and M.P. are the infectious diseases second opinion of the Italian National Center for Transplantation.*

### Financial support and sponsorship

*The authors received no financial support to produce this manuscript.*

### Conflicts of interest

*P.A.G. has the following conflict of interest: Consulting fees from Merck, Sharp & Dohme, Gilead Sciences, Takeda, Allovir; member of speakers’ bureau for Merck, Sharp & Dohme, Gilead Sciences, Takeda, Astra-Zeneca; M.P. has no conflicts of interest to declare. Please refer to the accompanying ICMJE disclosure forms for further details.*

## REFERENCES AND RECOMMENDED READING

Papers of particular interest, published within the annual period of review, have been highlighted as:

▪ of special interest
▪▪ of outstanding interest
